# μ-Oxalato-bis­[(2,2′-bipyridyl)­copper(II)] bis(perchlorate) dimethyl­formamide disolvate monohydrate

**DOI:** 10.1107/S1600536810031569

**Published:** 2010-08-18

**Authors:** Alexander N. Boyko, Matti Haukka, Irina A. Golenya, Svetlana V. Pavlova, Natalia I. Usenko

**Affiliations:** a Kiev National Taras Shevchenko University, Department of Chemistry, Volodymyrska str. 64, 01601 Kiev, Ukraine; b Department of Chemistry, University of Joensuu, PO Box 111, 80101, Joensuu, Finland

## Abstract

The title compound, [Cu_2_(C_2_O_4_)(C_10_H_8_N_2_)_4_](ClO_4_)_2_·2C_3_H_7_NO·H_2_O, contains doubly charged centrosymmetric dinuclear oxalato-bridged copper(II) complex cations, perchlorate anions, and DMF and water solvate mol­ecules. In the complex cation, the oxalate ligand is coordinated in a bis-bidentate bridging mode to the Cu atoms. Each Cu atom has a distorted tetra­gonal-bipyramidal environment, being coordinated by two N atoms of the two chelating bipy ligands and two O atoms of the doubly deprotonated oxalate anion. Pairs of perchlorate anions and water mol­ecules are linked into recta­ngles by O—H⋯O bonds in which the perchlorate O atoms act as acceptors and the water mol­ecules as donors. Methyl groups of the DMF solvent molecule are disordered over two sites with occupancies of 0.453 (7):0.547 (7), and the water molecule is half-occupied.

## Related literature

For use of oxalic acid and its derivatives in mol­ecular magnetism and supra­molecular chemistry, see: Kahn (1987 ▶); Ojima & Nonoyama (1988 ▶); Fritsky *et al.* (1998 ▶); Świątek-Kozłowska *et al.* (2000 ▶). For use of oxalic acid for the preparation of mixed-ligand polynuclear complexes, see: Strotmeyer *et al.* (2003 ▶). For related structures, see: Krämer & Fritsky (2000 ▶); Kovbasyuk *et al.* (2004 ▶); Wörl *et al.* (2005 ▶); Tomyn *et al.* (2007 ▶); Moroz *et al.* (2010 ▶). 
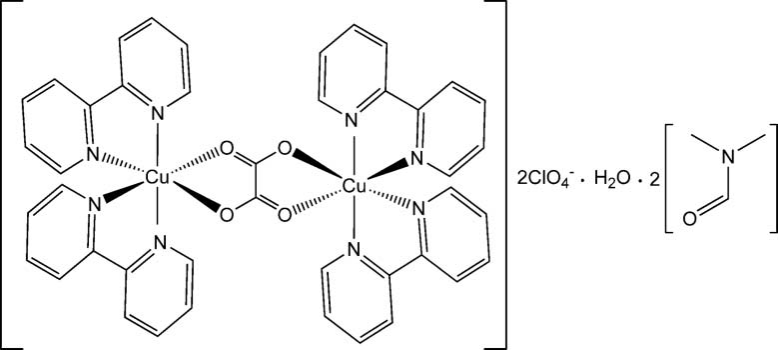


## Experimental

### 

#### Crystal data

[Cu_2_(C_2_O_4_)(C_10_H_8_N_2_)_4_](ClO_4_)_2_·2C_3_H_7_NO·H_2_O
*M* *_r_* = 1202.94
Triclinic, 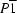

*a* = 9.6872 (5) Å
*b* = 11.0080 (8) Å
*c* = 12.2449 (5) Å
α = 97.928 (3)°
β = 99.565 (2)°
γ = 91.924 (2)°
*V* = 1273.16 (12) Å^3^
*Z* = 1
Mo *K*α radiation
μ = 1.02 mm^−1^
*T* = 100 K
0.23 × 0.12 × 0.08 mm

#### Data collection

Bruker Kappa APEXII DUO CCD diffractometer
Absorption correction: multi-scan (*SADABS*; Bruker, 2009 ▶) *T*_min_ = 0.802, *T*_max_ = 0.923
10051 measured reflections
4993 independent reflections
3955 reflections with *I* > 2σ(*I*)
*R*_int_ = 0.023

#### Refinement

*R*[*F* ^2^ > 2σ(*F* ^2^)] = 0.047
*wR*(*F* ^2^) = 0.129
*S* = 1.04
4993 reflections
359 parameters
27 restraints
H-atom parameters constrained
Δρ_max_ = 1.43 e Å^−3^
Δρ_min_ = −0.70 e Å^−3^

### 

Data collection: *APEX2* (Bruker, 2009 ▶); cell refinement: *SAINT* (Bruker, 2009 ▶); data reduction: *SAINT*; program(s) used to solve structure: *SHELXS97* (Sheldrick, 2008 ▶); program(s) used to refine structure: *SHELXL97* (Sheldrick, 2008 ▶); molecular graphics: *DIAMOND* (Bradenburg, 2006 ▶); software used to prepare material for publication: *SHELXL97*.

## Supplementary Material

Crystal structure: contains datablocks global, I. DOI: 10.1107/S1600536810031569/jh2192sup1.cif

Structure factors: contains datablocks I. DOI: 10.1107/S1600536810031569/jh2192Isup2.hkl

Additional supplementary materials:  crystallographic information; 3D view; checkCIF report

## Figures and Tables

**Table 1:** Hydrogen-bond geometry (Å, °)

*D*—H⋯*A*	*D*—H	H⋯*A*	*D*⋯*A*	*D*—H⋯*A*
O1*W*—H1*W*⋯O5^i^	0.91	2.45	3.311 (13)	159
O1*W*—H2*W*⋯O5	0.85	2.04	2.882 (14)	169

Symmetry code: (i) 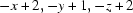.

**Fig. 1.**
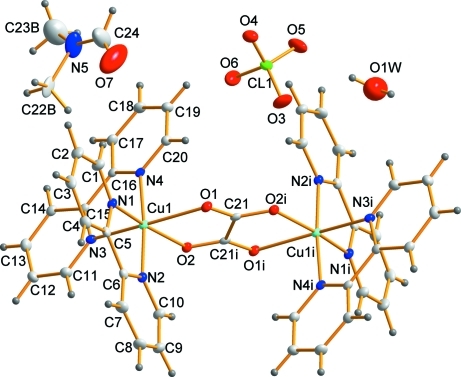
A view of compound (1), with displacement ellipsoids shown at the 30% probability level. H atoms are drawn as spheres of arbitrary radii. [Symmetry code: (i)1-x,1-y,1-z].

**Fig. 2.**
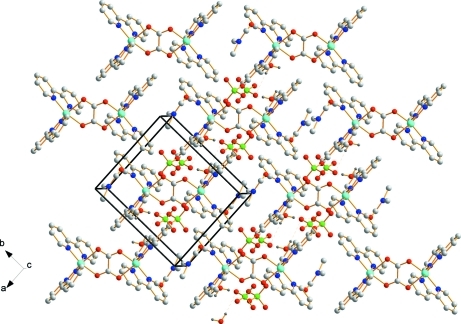
A packing diagram of the title compound. Hydrogen bonds are indicated by dashed lines. H atoms not involved in hydrogen bonding have been omitted for clarity.

## supplementary crystallographic information

### Comment

Oxalic acid and its amide derivatives are widely used in molecular magnetism and supramolecular chemistry for preparation of various bi- and polynuclear complexes as well as exchange clusters of high nuclearity (Kahn, 1987; Ojima & Nonoyama, 1988; Fritsky *et al.*, 1998; Świątek-Kozłowska *et al.*, 2000). Use of additional bridging ligands sometimes results in increase of nuclearity of the target mixed ligands compounds (Strotmeyer *et al.*, 2003). However, use of this synthetic strategy is often restricted by formation of complex species containing only one of the used bridging ligands. Herein we report a compound (I) isolated as a result of an attempt to obtain a mixed ligand complex containing both oxalate and pyridine-2-hydroxamate ligands.

In (I), the Cu atom has a distorted tetragonal-bipyramidal environment, with one oxygen atom of the oxalate (O2), three nitrogen atoms of the 2,2'-bipyridine ligand (N1, N2 and N4) occupying the base of the pyramid, and the second oxygen atom of the oxalate (O1) and one of the bipyridine nitrogen atoms (N3) in the apical positions (Fig. 1). The planar µ_4_-oxalato group lies in a center of symmetry and bridges the two copper atoms in a bis(chelating) mode, each copper atom being bound to two oxygens from the two different carboxylic groups. The Cu···Cu separation in the dimer is 5.6032 (9)Å which is slightly longer than the intermetallic separation observed in a related µ_4_-oxalato-bridged dicopper complex with (1-(pyridin-2-yl)ethylidene)hydrazine (5.449 (1) Å) (Tomyn *et al.*, 2007). The C-N and C-C bond lenths in the 2,2'-bipyridine ligands are normal for 2-substituted pyridine derivatives (Krämer *et al.*, 2000; Kovbasyuk *et al.*, 2004; Wörl *et al.*, 2005; Moroz *et al.*, 2010).

In the crystal packing, the dimeric complex cations are organized in layers disposed parallel to the xy plane. The neighboring cations are linked by stacking interactions between the pyridine rings (both along x and y directions) and by van der Waals forces. The perchlorate anions and solvate water molecules are disposed between the cationic layers. Two pairs of the translational perchlorate anions and water molecules form rectangles due to H-bonds where perchlorate O atoms act as acceptors and H_2_O molecules as donors (Fig. 2, Table 1).

### Experimental

Cu(ClO_4_)_2_.6H_2_O (0.371 g, 1 mmol) was dissolved in water (5 ml) and added to the dimethylformamide solution of pyridine-2-hydroxamic acid (0.138 g, 1 mmol) and 2,2'-bipyridine (0.156 g, 1 mmol), and then a powder of K_2_C_2_O_4_.H_2_O (0.092 g, 0.5 mmol) was added to the obtained solution. The resulting mixture was being stirred at 60 C° during 15 min and filtered. Turquoise crystals suitable for X-ray analysis were obtained by slow diffusion of diethyl ether vapour to the resulting solution at room temperature within 72 hours. They were filtered off and washed with diethyl ether. Yield: 57%.

### Refinement

Methyl groups of the dimethylformamide solvent molecule were disordered over two sites with occupancies 0.45/0.55. The N-C distances in the dimethylformamide molecules were restrained to to be similar and the anisotropic displacement parameters of the methyl carbons were constrained to be equal. The water of crystallization was refined with occupancy of 0.5. The H_2_O hydrogen atoms were located from the difference Fourier map but constrained to ride on their parent atom, with U_iso_ = 1.5 U_eq_(parent atom). Other hydrogen atoms were positioned geometrically and were also constrained to ride on their parent atoms, with C—H = 0.95-0.98 Å, and U_iso_ = 1.2-1.5 U_eq_(parent atom). The highest peak is located 0.58 Å from atom C22B and the deepest hole is located 0.60 Å from atom O7.

### Crystal data

**Table:**

[Cu_2_(C_2_O_4_)(C_10_H_8_N_2_)_4_](ClO_4_)_2_·2C_3_H_7_NO·H_2_O	*Z* = 1
*M**_r_* = 1202.94	*F*(000) = 618
Triclinic, *P*1	*D*_x_ = 1.569 Mg m^−^^3^
Hall symbol: -P 1	Mo *K*α radiation, λ = 0.71073 Å
*a* = 9.6872 (5) Å	Cell parameters from 3860 reflections
*b* = 11.0080 (8) Å	θ = 2.3–27.4°
*c* = 12.2449 (5) Å	µ = 1.02 mm^−^^1^
α = 97.928 (3)°	*T* = 100 K
β = 99.565 (2)°	Block, turquoise
γ = 91.924 (2)°	0.23 × 0.12 × 0.08 mm
*V* = 1273.16 (12) Å^3^	

### Data collection

**Table:**

Bruker Kappa APEXII DUO CCD diffractometer	4993 independent reflections
Radiation source: fine-focus sealed tube	3955 reflections with *I* > 2σ(*I*)
curved graphite crystal	*R*_int_ = 0.023
Detector resolution: 16 pixels mm^-1^	θ_max_ = 26.0°, θ_min_ = 1.7°
φ scans and ω scans with κ offset	*h* = −11→11
Absorption correction: multi-scan (*SADABS*; Bruker, 2009)	*k* = −13→13
*T*_min_ = 0.802, *T*_max_ = 0.923	*l* = −10→15
10051 measured reflections	

### Refinement

**Table:**

Refinement on *F*^2^	Primary atom site location: structure-invariant direct methods
Least-squares matrix: full	Secondary atom site location: difference Fourier map
*R*[*F*^2^ > 2σ(*F*^2^)] = 0.047	Hydrogen site location: inferred from neighbouring sites
*wR*(*F*^2^) = 0.129	H-atom parameters constrained
*S* = 1.04	*w* = 1/[σ^2^(*F*_o_^2^) + (0.0663*P*)^2^ + 2.0663*P*] where *P* = (*F*_o_^2^ + 2*F*_c_^2^)/3
4993 reflections	(Δ/σ)_max_ < 0.001
359 parameters	Δρ_max_ = 1.43 e Å^−^^3^
27 restraints	Δρ_min_ = −0.70 e Å^−^^3^

### Special details

**Table:**

Geometry. All esds (except the esd in the dihedral angle between two l.s. planes) are estimated using the full covariance matrix. The cell esds are taken into account individually in the estimation of esds in distances, angles and torsion angles; correlations between esds in cell parameters are only used when they are defined by crystal symmetry. An approximate (isotropic) treatment of cell esds is used for estimating esds involving l.s. planes.
Refinement. Refinement of F^2^ against ALL reflections. The weighted R-factor wR and goodness of fit S are based on F^2^, conventional R-factors R are based on F, with F set to zero for negative F^2^. The threshold expression of F^2^ > 2sigma(F^2^) is used only for calculating R-factors(gt) etc. and is not relevant to the choice of reflections for refinement. R-factors based on F^2^ are statistically about twice as large as those based on F, and R- factors based on ALL data will be even larger.

### Fractional atomic coordinates and isotropic or equivalent isotropic displacement parameters (Å^2^)

**Table:**

	*x*	*y*	*z*	*U*_iso_*/*U*_eq_	Occ. (<1)
Cu1	0.29558 (4)	0.30820 (4)	0.41931 (4)	0.01694 (15)	
Cl1	0.71283 (9)	0.33716 (9)	0.90518 (8)	0.0256 (2)	
O1	0.5313 (3)	0.3455 (2)	0.5049 (2)	0.0206 (6)	
O2	0.3249 (2)	0.4898 (2)	0.4286 (2)	0.0176 (5)	
O3	0.7943 (4)	0.3548 (3)	0.8209 (3)	0.0517 (9)	
O4	0.7375 (3)	0.2198 (3)	0.9423 (3)	0.0351 (7)	
O5	0.7470 (4)	0.4320 (3)	0.9976 (3)	0.0534 (10)	
O6	0.5683 (3)	0.3377 (4)	0.8567 (3)	0.0523 (10)	
O7	0.3074 (6)	0.0419 (7)	0.7731 (7)	0.138 (3)	
N1	0.3182 (3)	0.1260 (3)	0.4105 (3)	0.0199 (7)	
N2	0.3572 (3)	0.2758 (3)	0.2693 (3)	0.0176 (6)	
N3	0.0654 (3)	0.3071 (3)	0.3574 (3)	0.0190 (6)	
N4	0.2160 (3)	0.3330 (3)	0.5640 (3)	0.0177 (6)	
N5	0.0955 (5)	0.0617 (5)	0.7989 (5)	0.0720 (17)	
C1	0.2978 (4)	0.0562 (4)	0.4883 (4)	0.0293 (9)	
H1	0.2670	0.0932	0.5542	0.035*	
C2	0.3199 (4)	−0.0685 (4)	0.4759 (4)	0.0321 (10)	
H2	0.3052	−0.1160	0.5325	0.039*	
C3	0.3636 (4)	−0.1221 (3)	0.3802 (4)	0.0287 (9)	
H3	0.3776	−0.2076	0.3694	0.034*	
C4	0.3870 (4)	−0.0508 (3)	0.3001 (4)	0.0256 (8)	
H4	0.4179	−0.0863	0.2338	0.031*	
C5	0.3646 (3)	0.0738 (3)	0.3182 (3)	0.0186 (7)	
C6	0.3871 (3)	0.1588 (3)	0.2378 (3)	0.0180 (7)	
C7	0.4363 (4)	0.1248 (4)	0.1392 (3)	0.0269 (9)	
H7	0.4560	0.0418	0.1176	0.032*	
C8	0.4568 (4)	0.2125 (4)	0.0723 (3)	0.0292 (9)	
H8	0.4916	0.1906	0.0045	0.035*	
C9	0.4265 (4)	0.3320 (4)	0.1045 (3)	0.0267 (9)	
H9	0.4406	0.3939	0.0599	0.032*	
C10	0.3753 (4)	0.3601 (3)	0.2029 (3)	0.0233 (8)	
H10	0.3520	0.4421	0.2245	0.028*	
C11	−0.0050 (4)	0.2868 (4)	0.2522 (3)	0.0259 (8)	
H11	0.0470	0.2737	0.1928	0.031*	
C12	−0.1495 (4)	0.2839 (4)	0.2259 (4)	0.0279 (9)	
H12	−0.1961	0.2686	0.1503	0.033*	
C13	−0.2244 (4)	0.3038 (4)	0.3131 (4)	0.0275 (9)	
H13	−0.3239	0.3025	0.2979	0.033*	
C14	−0.1538 (4)	0.3256 (3)	0.4223 (3)	0.0210 (8)	
H14	−0.2038	0.3397	0.4829	0.025*	
C15	−0.0077 (4)	0.3266 (3)	0.4419 (3)	0.0177 (7)	
C16	0.0767 (4)	0.3479 (3)	0.5566 (3)	0.0177 (7)	
C17	0.0176 (4)	0.3815 (3)	0.6520 (3)	0.0233 (8)	
H17	−0.0802	0.3919	0.6458	0.028*	
C18	0.1027 (4)	0.3992 (4)	0.7553 (3)	0.0274 (9)	
H18	0.0641	0.4226	0.8209	0.033*	
C19	0.2450 (4)	0.3828 (4)	0.7632 (3)	0.0273 (9)	
H19	0.3052	0.3939	0.8339	0.033*	
C20	0.2973 (4)	0.3496 (3)	0.6654 (3)	0.0218 (8)	
H20	0.3947	0.3381	0.6703	0.026*	
C21	0.5591 (4)	0.4576 (3)	0.5219 (3)	0.0168 (7)	
C22A	0.0103 (10)	0.0664 (11)	0.7051 (11)	0.049 (2)	0.453 (7)
H22A	−0.0851	0.0770	0.7202	0.074*	0.453 (7)
H22B	0.0119	−0.0101	0.6540	0.074*	0.453 (7)
H22C	0.0402	0.1359	0.6707	0.074*	0.453 (7)
C23A	0.075 (2)	0.1244 (19)	0.9138 (14)	0.107 (4)	0.453 (7)
H23A	−0.0240	0.1429	0.9113	0.161*	0.453 (7)
H23B	0.1336	0.2010	0.9343	0.161*	0.453 (7)
H23C	0.1010	0.0697	0.9696	0.161*	0.453 (7)
C22B	0.0510 (8)	0.0191 (9)	0.6636 (8)	0.049 (2)	0.547 (7)
H22D	−0.0497	0.0275	0.6410	0.074*	0.547 (7)
H22E	0.0726	−0.0668	0.6447	0.074*	0.547 (7)
H22F	0.1036	0.0711	0.6241	0.074*	0.547 (7)
C23B	−0.0322 (15)	0.1000 (16)	0.8376 (15)	0.107 (4)	0.547 (7)
H23D	−0.1106	0.0875	0.7745	0.161*	0.547 (7)
H23E	−0.0206	0.1872	0.8694	0.161*	0.547 (7)
H23F	−0.0517	0.0512	0.8950	0.161*	0.547 (7)
C24	0.2302 (8)	0.0497 (10)	0.8262 (7)	0.100 (3)	
H24	0.2637	0.0475	0.9034	0.121*	
O1W	0.9215 (13)	0.6412 (13)	0.9750 (10)	0.119 (4)	0.50
H1W	1.0104	0.6270	0.9637	0.179*	0.50
H2W	0.8765	0.5737	0.9756	0.179*	0.50

### Atomic displacement parameters (Å^2^)

**Table:**

	*U*^11^	*U*^22^	*U*^33^	*U*^12^	*U*^13^	*U*^23^
Cu1	0.0187 (2)	0.0152 (2)	0.0193 (3)	0.00275 (16)	0.00795 (17)	0.00502 (17)
Cl1	0.0245 (5)	0.0333 (5)	0.0202 (5)	0.0044 (4)	0.0052 (4)	0.0060 (4)
O1	0.0211 (13)	0.0159 (13)	0.0269 (15)	0.0031 (10)	0.0067 (11)	0.0074 (11)
O2	0.0161 (12)	0.0166 (12)	0.0217 (14)	0.0036 (9)	0.0059 (10)	0.0043 (10)
O3	0.059 (2)	0.058 (2)	0.049 (2)	0.0059 (18)	0.0366 (18)	0.0171 (18)
O4	0.0452 (17)	0.0316 (16)	0.0297 (17)	0.0101 (13)	0.0065 (14)	0.0069 (13)
O5	0.091 (3)	0.0350 (18)	0.0285 (18)	0.0096 (18)	−0.0034 (18)	−0.0029 (15)
O6	0.0277 (16)	0.072 (3)	0.062 (2)	0.0046 (16)	−0.0021 (16)	0.035 (2)
O7	0.070 (4)	0.139 (6)	0.236 (9)	0.031 (4)	0.044 (5)	0.107 (6)
N1	0.0209 (15)	0.0146 (15)	0.0249 (17)	−0.0006 (12)	0.0043 (13)	0.0058 (13)
N2	0.0170 (14)	0.0155 (14)	0.0210 (16)	0.0020 (11)	0.0037 (12)	0.0039 (12)
N3	0.0193 (15)	0.0214 (16)	0.0181 (16)	0.0022 (12)	0.0057 (12)	0.0058 (13)
N4	0.0165 (14)	0.0169 (15)	0.0214 (17)	0.0007 (11)	0.0050 (12)	0.0070 (13)
N5	0.038 (3)	0.060 (3)	0.117 (5)	0.011 (2)	0.032 (3)	−0.015 (3)
C1	0.038 (2)	0.023 (2)	0.030 (2)	−0.0007 (17)	0.0105 (18)	0.0074 (17)
C2	0.039 (2)	0.021 (2)	0.036 (3)	−0.0037 (17)	0.0019 (19)	0.0133 (18)
C3	0.031 (2)	0.0142 (18)	0.037 (2)	0.0020 (16)	−0.0067 (18)	0.0056 (17)
C4	0.0266 (19)	0.0194 (19)	0.029 (2)	0.0046 (15)	0.0007 (17)	−0.0002 (16)
C5	0.0144 (16)	0.0192 (18)	0.0200 (19)	−0.0001 (13)	−0.0033 (14)	0.0030 (15)
C6	0.0130 (16)	0.0188 (18)	0.0208 (19)	0.0018 (13)	0.0003 (14)	0.0006 (15)
C7	0.032 (2)	0.025 (2)	0.024 (2)	0.0060 (16)	0.0081 (17)	−0.0021 (16)
C8	0.032 (2)	0.036 (2)	0.021 (2)	0.0026 (18)	0.0107 (17)	0.0004 (18)
C9	0.029 (2)	0.028 (2)	0.025 (2)	−0.0008 (16)	0.0087 (17)	0.0088 (17)
C10	0.0278 (19)	0.0192 (18)	0.025 (2)	0.0029 (15)	0.0094 (16)	0.0057 (16)
C11	0.0257 (19)	0.030 (2)	0.023 (2)	0.0024 (16)	0.0068 (16)	0.0051 (17)
C12	0.027 (2)	0.032 (2)	0.024 (2)	−0.0016 (16)	−0.0008 (16)	0.0056 (17)
C13	0.0189 (18)	0.030 (2)	0.033 (2)	0.0015 (16)	0.0027 (16)	0.0049 (18)
C14	0.0208 (17)	0.0181 (18)	0.026 (2)	0.0016 (14)	0.0077 (15)	0.0051 (15)
C15	0.0198 (17)	0.0132 (16)	0.0215 (19)	0.0017 (13)	0.0072 (15)	0.0032 (14)
C16	0.0188 (17)	0.0148 (17)	0.0213 (19)	−0.0001 (13)	0.0068 (14)	0.0055 (14)
C17	0.0205 (18)	0.0253 (19)	0.026 (2)	0.0008 (15)	0.0103 (16)	0.0030 (16)
C18	0.028 (2)	0.034 (2)	0.023 (2)	0.0019 (17)	0.0125 (17)	0.0038 (17)
C19	0.026 (2)	0.035 (2)	0.022 (2)	−0.0014 (17)	0.0053 (16)	0.0073 (17)
C20	0.0188 (17)	0.0252 (19)	0.023 (2)	0.0006 (15)	0.0053 (15)	0.0062 (16)
C21	0.0198 (17)	0.0185 (18)	0.0159 (18)	0.0044 (14)	0.0093 (14)	0.0070 (14)
C22A	0.011 (3)	0.039 (4)	0.098 (6)	0.002 (3)	0.005 (3)	0.021 (4)
C23A	0.083 (6)	0.126 (7)	0.107 (7)	0.019 (5)	0.019 (5)	−0.007 (6)
C22B	0.011 (3)	0.039 (4)	0.098 (6)	0.002 (3)	0.005 (3)	0.021 (4)
C23B	0.083 (6)	0.126 (7)	0.107 (7)	0.019 (5)	0.019 (5)	−0.007 (6)
C24	0.050 (4)	0.178 (10)	0.066 (5)	−0.029 (5)	−0.004 (4)	0.017 (5)
O1S	0.095 (8)	0.155 (12)	0.110 (10)	−0.004 (8)	0.015 (7)	0.032 (9)

### Geometric parameters (Å, °)

**Table:**

Cu1—O2	1.995 (2)	C8—H8	0.9500
Cu1—N2	2.015 (3)	C9—C10	1.378 (5)
Cu1—N1	2.015 (3)	C9—H9	0.9500
Cu1—N4	2.036 (3)	C10—H10	0.9500
Cu1—N3	2.232 (3)	C11—C12	1.381 (5)
Cu1—O1	2.346 (3)	C11—H11	0.9500
Cl1—O5	1.417 (3)	C12—C13	1.383 (6)
Cl1—O6	1.428 (3)	C12—H12	0.9500
Cl1—O3	1.428 (3)	C13—C14	1.382 (6)
Cl1—O4	1.442 (3)	C13—H13	0.9500
O1—C21	1.236 (4)	C14—C15	1.395 (5)
O2—C21^i^	1.265 (4)	C14—H14	0.9500
O7—C24	1.065 (9)	C15—C16	1.486 (5)
N1—C1	1.339 (5)	C16—C17	1.395 (5)
N1—C5	1.348 (5)	C17—C18	1.375 (6)
N2—C10	1.340 (5)	C17—H17	0.9500
N2—C6	1.348 (4)	C18—C19	1.384 (5)
N3—C11	1.339 (5)	C18—H18	0.9500
N3—C15	1.346 (5)	C19—C20	1.385 (5)
N4—C20	1.340 (5)	C19—H19	0.9500
N4—C16	1.354 (4)	C20—H20	0.9500
N5—C22A	1.313 (11)	C21—O2^i^	1.265 (4)
N5—C24	1.305 (9)	C21—C21^i^	1.573 (7)
N5—C23B	1.463 (14)	C22A—H22A	0.9800
N5—C23A	1.527 (14)	C22A—H22B	0.9800
N5—C22B	1.631 (11)	C22A—H22C	0.9800
C1—C2	1.387 (6)	C23A—H23A	0.9800
C1—H1	0.9500	C23A—H23B	0.9800
C2—C3	1.375 (6)	C23A—H23C	0.9800
C2—H2	0.9500	C22B—H22D	0.9800
C3—C4	1.379 (6)	C22B—H22E	0.9800
C3—H3	0.9500	C22B—H22F	0.9800
C4—C5	1.390 (5)	C23B—H23D	0.9800
C4—H4	0.9500	C23B—H23E	0.9800
C5—C6	1.485 (5)	C23B—H23F	0.9800
C6—C7	1.380 (5)	C24—H24	0.9500
C7—C8	1.379 (6)	O1W—H1W	0.9100
C7—H7	0.9500	O1W—H2W	0.8500
C8—C9	1.377 (6)		
			
O2—Cu1—N2	92.86 (11)	C8—C9—C10	118.6 (4)
O2—Cu1—N1	165.61 (11)	C8—C9—H9	120.7
N2—Cu1—N1	80.78 (12)	C10—C9—H9	120.7
O2—Cu1—N4	89.79 (11)	N2—C10—C9	122.3 (4)
N2—Cu1—N4	174.69 (12)	N2—C10—H10	118.8
N1—Cu1—N4	97.65 (12)	C9—C10—H10	118.8
O2—Cu1—N3	93.94 (10)	N3—C11—C12	123.2 (4)
N2—Cu1—N3	97.73 (11)	N3—C11—H11	118.4
N1—Cu1—N3	99.68 (12)	C12—C11—H11	118.4
N4—Cu1—N3	77.48 (11)	C11—C12—C13	118.1 (4)
O2—Cu1—O1	77.12 (9)	C11—C12—H12	121.0
N2—Cu1—O1	89.30 (11)	C13—C12—H12	121.0
N1—Cu1—O1	89.83 (10)	C14—C13—C12	119.7 (4)
N4—Cu1—O1	95.78 (10)	C14—C13—H13	120.2
N3—Cu1—O1	168.94 (10)	C12—C13—H13	120.2
O5—Cl1—O6	110.2 (2)	C13—C14—C15	118.8 (4)
O5—Cl1—O3	110.4 (2)	C13—C14—H14	120.6
O6—Cl1—O3	107.9 (2)	C15—C14—H14	120.6
O5—Cl1—O4	109.5 (2)	N3—C15—C14	121.6 (3)
O6—Cl1—O4	108.4 (2)	N3—C15—C16	115.9 (3)
O3—Cl1—O4	110.2 (2)	C14—C15—C16	122.5 (3)
C21—O1—Cu1	108.6 (2)	N4—C16—C17	121.1 (3)
C21^i^—O2—Cu1	119.4 (2)	N4—C16—C15	116.2 (3)
C1—N1—C5	118.8 (3)	C17—C16—C15	122.7 (3)
C1—N1—Cu1	126.4 (3)	C18—C17—C16	119.2 (3)
C5—N1—Cu1	114.7 (2)	C18—C17—H17	120.4
C10—N2—C6	119.1 (3)	C16—C17—H17	120.4
C10—N2—Cu1	125.8 (3)	C17—C18—C19	119.8 (4)
C6—N2—Cu1	115.0 (2)	C17—C18—H18	120.1
C11—N3—C15	118.6 (3)	C19—C18—H18	120.1
C11—N3—Cu1	129.3 (3)	C20—C19—C18	118.3 (4)
C15—N3—Cu1	112.0 (2)	C20—C19—H19	120.9
C20—N4—C16	119.0 (3)	C18—C19—H19	120.9
C20—N4—Cu1	122.7 (2)	N4—C20—C19	122.7 (3)
C16—N4—Cu1	117.9 (2)	N4—C20—H20	118.7
C22A—N5—C24	134.7 (8)	C19—C20—H20	118.7
C22A—N5—C23B	78.1 (9)	O1—C21—O2^i^	125.4 (3)
C24—N5—C23B	146.3 (9)	O1—C21—C21^i^	117.5 (4)
C22A—N5—C23A	125.4 (9)	O2^i^—C21—C21^i^	117.1 (4)
C24—N5—C23A	96.1 (9)	N5—C22A—H22A	109.4
C23B—N5—C23A	49.9 (9)	N5—C22A—H22B	109.5
C24—N5—C22B	108.0 (6)	H22A—C22A—H22B	109.5
C23B—N5—C22B	105.5 (9)	N5—C22A—H22C	109.5
C23A—N5—C22B	154.7 (8)	H22A—C22A—H22C	109.5
N1—C1—C2	122.2 (4)	H22B—C22A—H22C	109.5
N1—C1—H1	118.9	N5—C23A—H23A	109.5
C2—C1—H1	118.9	N5—C23A—H23B	109.4
C3—C2—C1	118.8 (4)	H23A—C23A—H23B	109.5
C3—C2—H2	120.6	N5—C23A—H23C	109.5
C1—C2—H2	120.6	H23A—C23A—H23C	109.5
C2—C3—C4	119.6 (4)	H23B—C23A—H23C	109.5
C2—C3—H3	120.2	N5—C22B—H22D	109.5
C4—C3—H3	120.2	N5—C22B—H22E	109.5
C3—C4—C5	118.7 (4)	H22D—C22B—H22E	109.5
C3—C4—H4	120.6	N5—C22B—H22F	109.5
C5—C4—H4	120.6	H22D—C22B—H22F	109.5
N1—C5—C4	121.8 (3)	H22E—C22B—H22F	109.5
N1—C5—C6	115.0 (3)	N5—C23B—H23D	109.5
C4—C5—C6	123.2 (4)	N5—C23B—H23E	109.5
N2—C6—C7	121.2 (3)	H23D—C23B—H23E	109.5
N2—C6—C5	114.4 (3)	N5—C23B—H23F	109.4
C7—C6—C5	124.4 (3)	H23D—C23B—H23F	109.5
C8—C7—C6	119.3 (4)	H23E—C23B—H23F	109.5
C8—C7—H7	120.3	O7—C24—N5	128.6 (9)
C6—C7—H7	120.3	O7—C24—H24	115.7
C9—C8—C7	119.4 (4)	N5—C24—H24	115.7
C9—C8—H8	120.3	H1W—O1W—H2W	110.2
C7—C8—H8	120.3		

Symmetry codes: (i) −*x*+1, −*y*+1, −*z*+1.

### Hydrogen-bond geometry (Å, °)

**Table:**

*D*—H···*A*	*D*—H	H···*A*	*D*···*A*	*D*—H···*A*
O1W—H1W···O5^ii^	0.91	2.45	3.311 (13)	159.
O1W—H2W···O5	0.85	2.04	2.882 (14)	169.

Symmetry codes: (ii) −*x*+2, −*y*+1, −*z*+2.
